# Heyde syndrome: Experiences with the use of semiautomatic vWF multimer analysis for diagnosis and TAVI for treatment – case report

**DOI:** 10.1097/MD.0000000000042486

**Published:** 2025-05-16

**Authors:** Boris Focko, Zuzana Miertová, Ingrid Škorňová, Martin Hudec, Martin Jozef Péč, Jakub Jurica, Marek Cingel, Tomáš Bolek, Juraj Sokol, Petra Poliačiková, Marián Mokáň, Matej Samoš

**Affiliations:** a Department of Internal Medicine I, Jessenius Faculty of Medicine in Martin, Comenius University in Bratislava and University Hospital Martin, Martin, Slovakia; b Department of Hematology and Transfusiology, National Centre of Hemostasis and Thrombosis, Jessenius Faculty of Medicine in Martin, Comenius University in Bratislava and University Hospital Martin, Martin, Slovakia; c Department of Acute and Interventional Cardiology, Central Slovak Institute of Heart and Vascular Diseases (SÚSCCH), a.s., Banská Bystrica, Slovakia.

**Keywords:** acquired von Willebrand syndrome, aortic stenosis, Heyde syndrome, semiautomatic von Willebrand factor multimer analysis

## Abstract

**Rationale:**

Aortic stenosis (AS) is currently the most frequent valve disorder. In addition, the angiodysplasias are the most common vascular malformations of the gastrointestinal tract. Heyde syndrome (HS) is a rare disease which links these 2 pathological conditions.

**Patient concerns:**

Currently, there are no clearly defined guidelines for the confirmation of HS diagnosis and for timing of aortic valve replacement in patients with confirmed HS, despite the fact that HS is connected with increased mortality and the need for numerous rehospitalizations and multiple blood transfusions. Therefore, the aim of this study was to report (our) first experiences with novel diagnostic method for acquired von Willebrand (vW) syndrome and transcatheter aortic valve replacement (TAVR) for treatment of HS.

**Diagnoses:**

We report a case of a 68-year-old man who was diagnosed with HS with the use of semiautomatic vW factor multimer analysis.

**Interventions:**

A successful TAVR was used for treatment of AS.

**Outcomes:**

After the interventional treatment of AS, the patient did not have a recurrence of the anemic syndrome.

**Lessons:**

This is a unique case of a patient with HS in whom semiautomatic vW factor multimer analysis was used for diagnosis of acquired vW syndrome together with TAVR procedure for HS treatment.

## 1. Introduction

Heyde syndrome (HS) is a relatively rare condition in which aortic stenosis (AS) and bleeding from angiodysplasia of the gastrointestinal tract (GIT) occur simultaneously. An acquired von Willebrand (vW) syndrome due to pathological deformation of vW factor (vWF) multimers on the stenotic aortic valve is considered to be the most probable pathological mechanism of its development. The reported prevalence of HS is 1% to 3% in patients with AS, while the prevalence of AS reaches 2% to 7% in patients over 65 years of age.^[1]^ On the other hand, angiodysplasias are the most common vascular malformations in the GIT causing various severe bleedings – from positive occult bleeding to massive bleedings with a hemodynamic response. The mortality of patients with HS reaches approximately 7%, not to mention the large amount of blood products required to replace the anemic syndrome in these patients.^[2]^

We would like to present a case of a patient with paradoxical low flow/low gradient (LF/LG) severe AS and HS in whom acquired von Willebrand syndrome was confirmed by semiautomatic vWF multimer analysis and who was successfully treated with transcatheter aortic valve implantation (TAVI).

## 2. Case

We present the case of a 68-year-old Caucasian man examined at the emergency department for weakness, exertional dyspnea, and vertigo without syncope. His personal history includes hypertension, type 2 diabetes, dyslipidaemia, diverticulosis of the large intestine (without any signs of bleeding) and history of repeated symptomatic anemia requiring in-hospital stays and red blood cells substitution. Anaemia has previously been caused by bleeding from internal hemorrhoids that were ligated and resected by Milligan-Morgan hemorrhoidectomy, and twice by acute bleeding from a Dieulafoy lesion subsequently treated with hemoclips. The patient was taking a beta-blocker and an angiotensin-converting enzyme inhibitor as part of hypertension treatment, a proton pump inhibitor, fenofibrate for dyslipidaemia, iron replacement, and insulin therapy. The rectal examination was without any traces of fresh blood on the glove, the abdomen was painless on palpation, without palpable resistance, and the rest of physical examination was also without pathological findings, except for the presence of a systolic murmur in the precordium of 3/6 intensity and with propagation to the carotid arteries. The initial electrocardiogram indicated left ventricular hypertrophy, without any ischemic changes, and a chest X-ray was also performed with findings of aortosclerosis without cardiomegaly.

The initial laboratory examination of the blood count and blood serum showed microcytic hypochromic anemia (with a hemoglobin concentration of 81 g/L) and a hematocrit of 0.27. The platelet count was 196 × 10^9^/L, creatinine level was 105 µmol/L, the estimated glomerular filtration rate was 63 mL/min/1.73m^2^ and N-terminal pro-B natriuretic peptide (NT-proBNP) levels were 143.7 ng/L. Troponin was negative. The test for occult bleeding was repeatedly positive.

**Figure 1.**
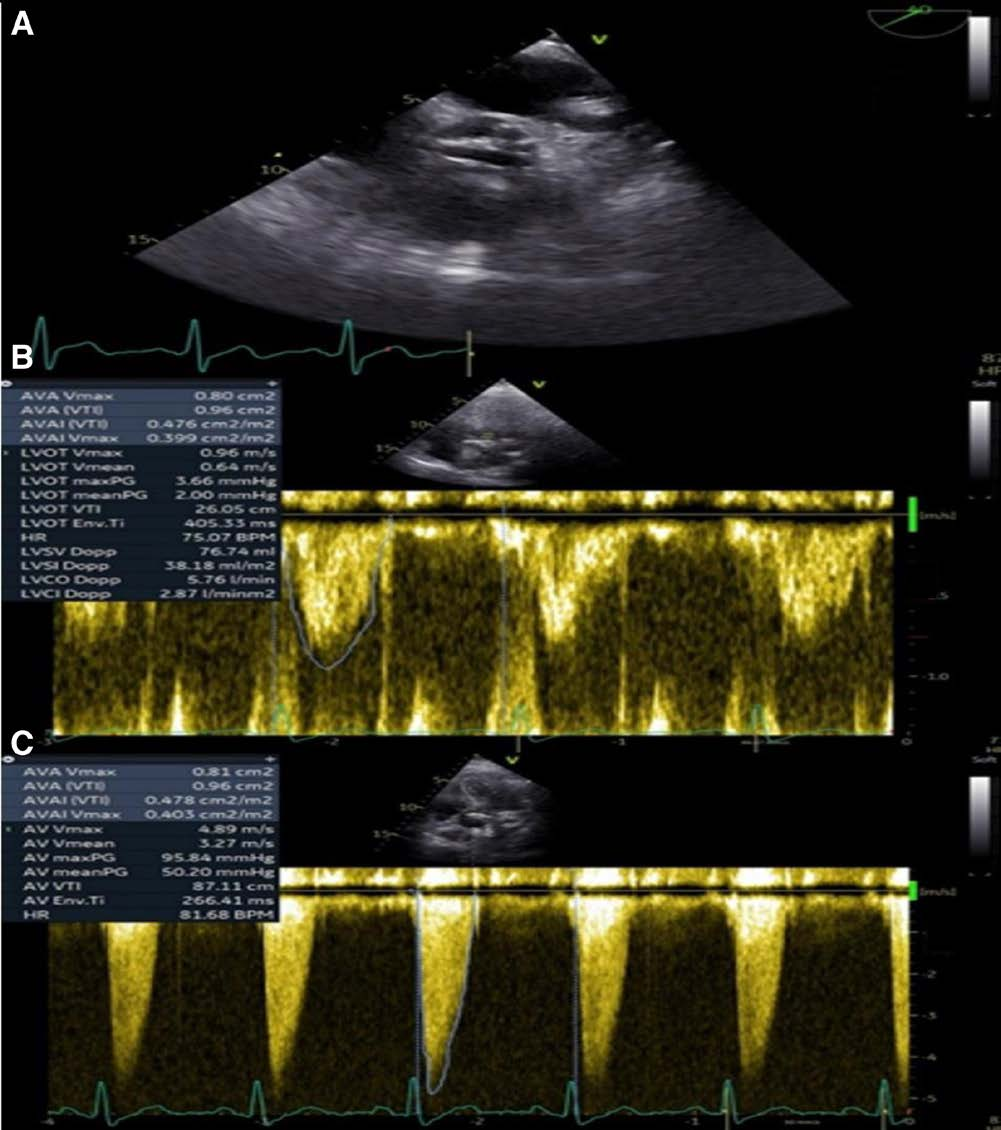
Severe aortic stenosis on transesophageal echocardiography (A), pulsed Doppler echocardiography during dobutamine loading focusing on blood flow in the left ventricular outflow tract (B), continuous Doppler echocardiography during dobutamine loading focused on the aortic valve (C).

Standard endoscopic examinations such as esophagogastroduodenoscopy and colonoscopy did not reveal any possible source of blood loss (no signs of active or past bleeding were found), even enterography with computed tomography (CT enterography) and endoscopic enteroscopy were not helpful in the diagnostic process of recurrent anemia. Because of the systolic murmur, a transthoracic echocardiographic examination was performed. A severe LF/LG AS was found (aortic valve area/AVA/0.9 cm^2^, maximum peak velocity 3.6 m/s, mean gradient 35 mm Hg). In addition, echocardiography also revealed hypertrophy of the left ventricle with type I diastolic dysfunction, without regional wall motion abnormalities or systolic left ventricular dysfunction. For a definitive confirmation of significant AS, transesophageal echocardiography (Fig. 1) and dobutamine stress echocardiography (Fig. 1) were performed, which confirmed severe paradoxical LF/LG AS.

**Figure 2.**
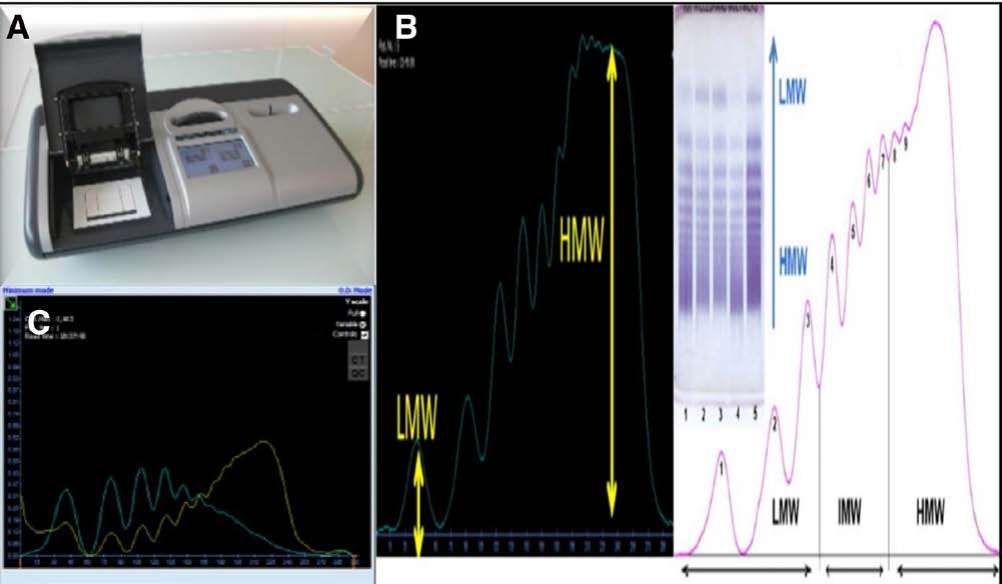
Analysis of vWF multimers with the Hydrasys 2 scan densitometric device: example of device (A), example of a study protocol (B), study protocol of our patient confirming acquired von Willebrand syndrome/blue curve – patient sample, yellow curve – control (C). vWF = von Willebrand factor.

Due to the hypothesis that AS was the cause of recurrent anemia, a hematologist was consulted with a recommendation to examine the vWF levels and platelet function testing.^[3]^ The vWF antigen levels were normal, but the analysis of platelet functions (their adhesion and aggregation) using a membrane with collagen/adrenaline and collagen/adenosine diphosphate (platelet function analyzer with collagen/epinephrine and collagen/adenosine diphosphate/ADP/membrane – PFA-COL/EPI and PFA-COL/ADP) indicated a defect in platelet functions. The binding of vWF to platelets in the presence of ristocetin as an agglutinating agent was reduced, while the analysis of vWF multimers using a semiautomatic Hydrasys 2 device (Sebia, Lisses, France, Fig. 2) and densitometric evaluation demonstrated a reduced distribution of high molecular weight vWF multimers, which indicated acquired von Willebrand syndrome, fulfilling the criteria for the diagnosis of HS (Fig. 2).^[4]^

**Figure 3.**
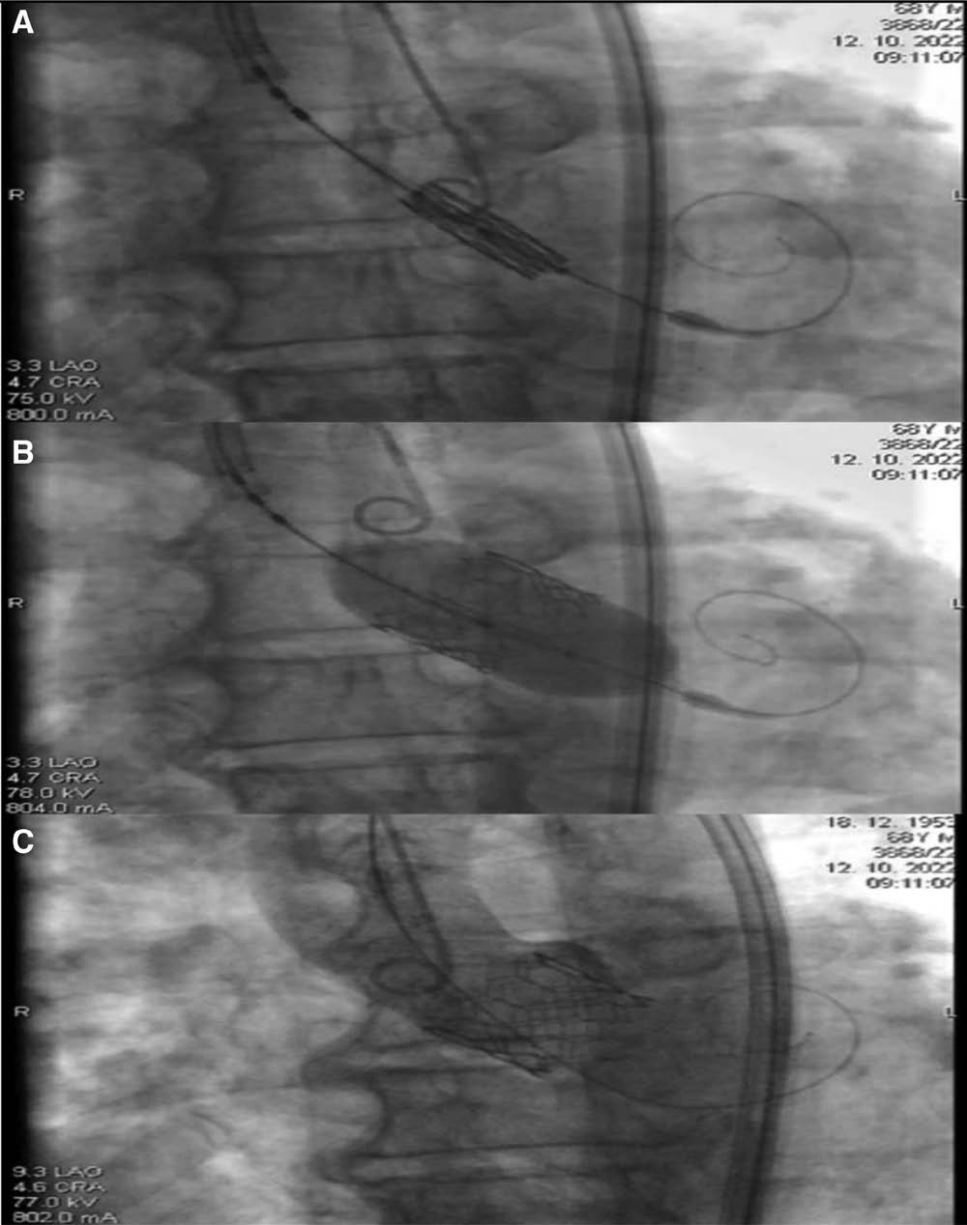
Transfemoral TAVI – positioning of the bioprosthesis in the stenotic native valve (A), Expanding the bioprosthesis with a balloon (B), Suppression of the native valve by a new bioprosthesis that immediately assumes a role in the cardiac cycle (C). TAVI = transcatheter aortic valve implantation.

**Figure 4.**
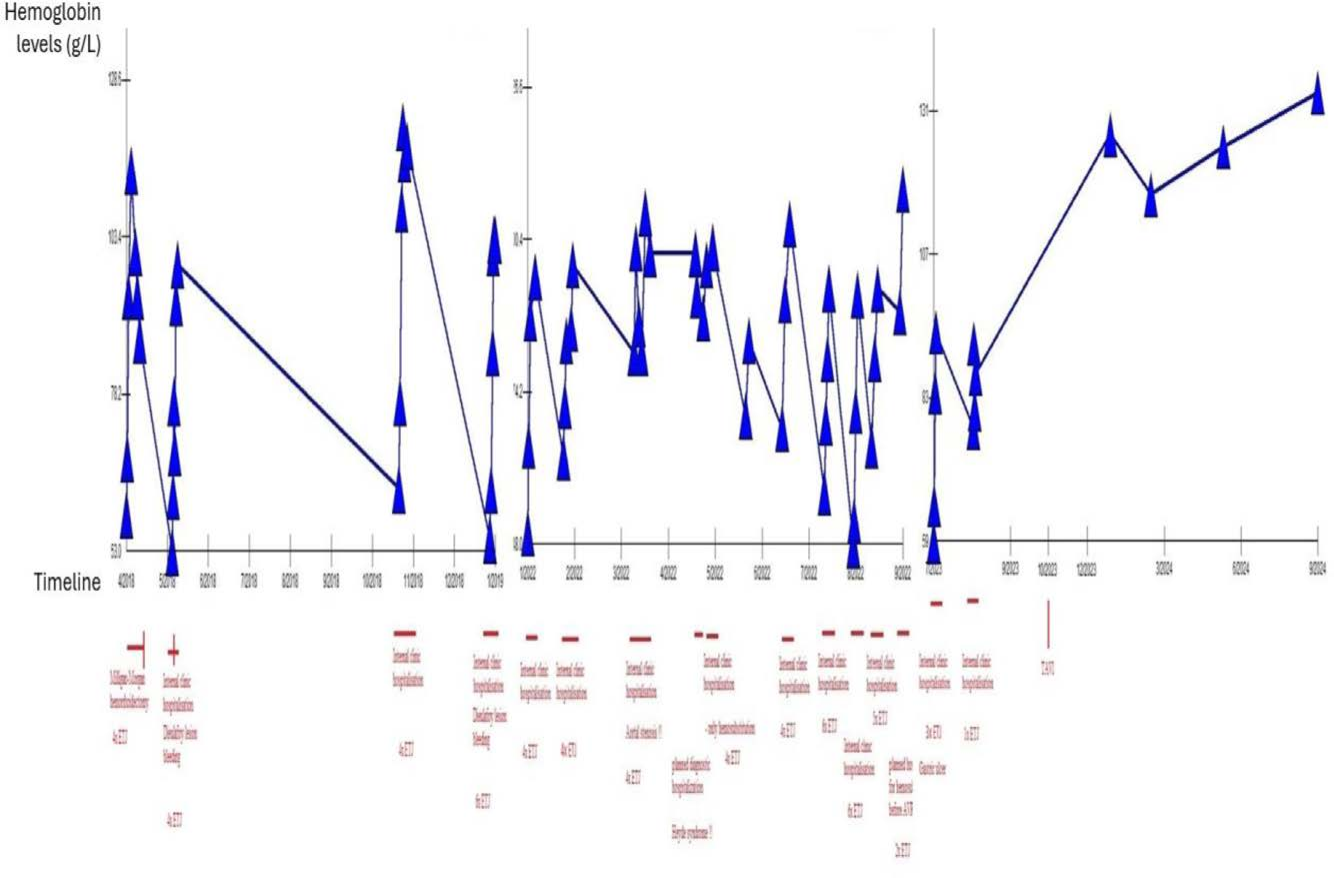
The dynamics of hemoglobin levels during treatment of various etiologies and iron/red blood cell concentrates supplementation and after TAVI procedure. TAVI = transcatheter aortic valve implantation.

Subsequently, the patient was referred for a surgical/interventional treatment, and following a heart team discussion, a transcatheter approach for aortic valve replacement was chosen. A balloon-expandable valve was implanted (Fig. 3); however, from the diagnosis of HS to the time of TAVI, a total number of 61 transfusion units of erythrocytes were needed for symptomatic anemic syndrome. The dynamics in hemoglobin levels is reported in Figure 3 (Fig. 4). The delay in performing TAVI procedure was caused by a long waiting list and the fact that HS is not an indication for an acute procedure. After TAVI, there was no need for patient readmission for symptomatic anemia during 2 years of clinical follow-up.

## 3. Discussion

Looking at the available clinical data so far, one can conclude that the majority of reported HS patients had high gradient (mean gradient ≥ 40 mm Hg) aortic stenoses, and the diagnosis of HS was based on fulfilling the trias of diagnostic criteria consisting of the presence of severe AS, gastrointestinal bleeding and the presence (or suggested existence) of gastrointestinal angiodysplasias,^[5,6]^ without exact confirmation of acquired vW disease. In contrast to previously published cases, the diagnosis of acquired vW syndrome in our patient was confirmed with novel semiautomatic vW multimer analysis method. One must remember that vW factor plasma levels (assessed by antigen-detection based assays) might be “normal” in patients with acquired vW disease,^[7,8]^ and that the diagnosis must be determined with vW multimer analysis. However, this is traditionally limited by difficulties associated with the traditional “manual” method of vW multimer assessment. On the contrary, semiautomatic method is based on a modified electrophoretic analysis, which is performed with a semiautomatic device Hydrasys 2 (Sebia, Lisses, France, Fig. 2). Preprepared 2% agarose gels are used for this analysis, followed by direct immunofixation and visualization with an antibody labeled with peroxidase. In the next step, the stained gels with vWF multimers are visually inspected and scanned using software. Finally, the densitometric graph of each sample is qualitatively and quantitatively evaluated in comparison with the control sample, which is examined on the same gel. The intensity of individual peaks during densitometric examination is directly related to the concentration of vWF multimers (Fig. 2).^[9]^ Another possibility is the use of the examination of ristocetin cofactor activity of vWF, which tends to be significantly reduced, and is much more specific. This assay primarily detects high molecular weight multimers and is based on the ability of vWF to bind to platelets in the presence of ristocetin as an agglutinating agent. The collagen-binding activity of von Willebrand factor is also sensitive to the deficiency of large multimers, and is reduced in type 2A. In our patient, the binding of vWF to platelets in the presence of ristocetin as an agglutinating agent was reduced, confirming the diagnosis of acquired vW disease.

Unlike the majority of so far published cases,^[2–8]^ in which HS developed in the settings of high gradient AS, our patient with HS had a LF/LG AS. In fact, this seems to be the first case of HS associated with LF/LG AS. As previously mentioned, the most probable mechanism of HS development is the pathological deformation of vWF multimers on the stenotic aortic valve.^[9,10]^ Considering this mechanism (the degeneration of valve itself), it is not surprising that HS can develop also in the absence of high flow velocities on aortic valve (high gradient AS). Our presented case points to the fact that one should consider HS as a cause of GI bleeding also in those patients who have LF/LG AS.

Symptomatic treatment of HS includes iron replacement, either oral or parenteral, red blood cell transfusions and haemostyptics for active bleeding from the gastrointestinal tract, fibroscopic examinations are indicated for acute heavy bleeding. However, the only causal therapy for HS is aortic valve replacement. This can be performed by open surgery, replacing the aortic valve by a sternotomy approach, in which younger patients and those with fewer comorbidities are preferred. Elderly, high-risk patients are indicated for TAVI.^[6]^ However, it is not entirely clear whether TAVI is the appropriate treatment for HS.^[11]^ In a previous study, Wang et al^[12]^ reviewed international case reports of patients with HS and identified 31 cases of patients treated with TAVI. All these patients experienced repeated bleeding complications after pharmacological and endoscopic therapy in a similar way to our patient. TAVI lead to improved symptoms in all of the treated patients. In our case, the successful TAVI procedure lead to significant improvement of GI symptoms, with no repeated need for in-hospital admission during 2 years of clinical follow-up. These observations probably demonstrate that TAVI is a safe and effective therapeutic option for HS patients, who are not suitable for surgical aortic valve replacement. Nevertheless, as current evidence is limited to 32 published clinical cases so far (including our patient), more evidence from clinical studies will be needed to form final conclusions. While awaiting TAVI, up to 61 units of erythrocyte transfusions were required, leading to the question of whether patients with HS should be prioritized for exchange of the aortic valve, and this issue should be also addressed in future studies.

Summarizing the outcomes of our study, this study confirmed the clinical usefulness of novel semiautomatic method of vW multimer assessment with a modified electrophoretic analysis on a semiautomatic device Hydrasys 2. This method can be used effectively used for the confirmation of acquired vW syndrome in patients with suspected HS. Second, we confirmed that HS can develop also in the settings of (paradoxical) LF/LG AS, and that one should think on this rare disease also in LF/LG AS who develop gastrointestinal hemorrhage. Finally, we confirmed that TAVI can be effectively used as a treatment modality for HS, especially in those patients who are not suitable for surgical aortic valve replacement. However, several issues remains uncertain, mostly the fact when should we time aortic valve replacement (urgently vs electively) for symptomatic HS.

## 4. Limitations

There were several limitations of our analysis, which should be definitely taken into account when the results of our study are interpreted. First, this is a single case report, and therefore the study outcomes need to be confirmed in larger cohorts of patients. Second, although effective (and more user-friendly compared to traditional manual method), the novel semiautomatic method for vWF multimer analysis has still a limited availability (only a few laboratories dedicated on the diagnosis of vW syndrome have the ability to use this method), and the method itself needs an experienced laboratory staff to be performed. These facts limit its use in current clinical practice. Finally, as already mentioned, the current evidence for the use of TAVI procedure for the treatment of symptomatic HS is limited to 32 published clinical cases so far, and more evidence from clinical studies will be needed to form final recommendations for the use of TAVI in HS indication.

## Author contributions

**Conceptualization:** Martin Hudec, Tomáš Bolek, Matej Samoš.

**Data curation:** Martin Hudec, Jakub Jurica, Petra Poliačiková.

**Formal analysis:** Martin Jozef Péč, Marek Cingel.

**Investigation:** Boris Focko, Zuzana Miertová, Ingrid Škorňová, Martin Hudec, Martin Jozef Pec, Jakub Jurica, Marek Cingel, Tomáš Bolek, Petra Poliačiková, Matej Samoš.

**Methodology:** Ingrid Škorňová, Tomáš Bolek, Matej Samoš.

**Supervision:** Martin Hudec, Tomáš Bolek, Juraj Sokol, Marián Mokáň, Matej Samoš.

**Writing – original draft:** Boris Focko, Zuzana Miertová, Jakub Jurica.

**Writing – review & editing:** Juraj Sokol, Marián Mokáň, Matej Samoš.
